# Prostate Cancer – Local Treatment after Radiorecurrence: Surgery - Back to the future?

**DOI:** 10.1590/S1677-5538.IBJU.2018.03.04

**Published:** 2018

**Authors:** Leonardo O. Reis, Paul L. Nguyen

**Affiliations:** 1 Departamento de Oncologia Urológica (UroScience), Pontifícia Universidade Católica de Campinas, PUC - Campinas, SP, Brasil; 2 Universidade Estadual de Campinas, UNICAMP, Campinas, SP, Brasil; 3 Department of Radiation Oncology, Dana-Farber Cancer Institute and Brigham and Women's Hospital, Harvard Medical School, Boston, MA, USA

**Keywords:** Prostatic Neoplasms, Salvage Therapy, Radiation

As radiotherapy advances, inadequate deliveries decrease and salvage radical prostatectomy, when necessary, tends to be less challenging with improving oncological and functional results. It treats cancer foci in regions such as apex or periurethral tissue, often spared in ablative approaches to minimize side effects; adds the clear goal of an undetectable PSA; and the opportunity for pelvic lymph-node dissection to potentially treat loco regional micrometastasis.

Up to 50% of men may develop recurrence after definitive radiation for prostate cancer, and about one-third of these men will have a biopsy-proven local radiorecurrence, which occurs due to either inadequate delivery of the prescribed radiation dose or inherent biologic resistance of prostate cancer clones to radiation. Standard of care for these patients has been classically observation followed by androgen deprivation (ADT), which adds substantial morbidity and doesn't offer the possibility of cure.

In selected patients, radical prostatectomy, brachytherapy, cryotherapy, and high-intensity focused ultrasound (HiFU) offer reasonable cancer control outcomes without the need for salvage ADT, which is reserved for situations of clear evidence of systemic disease, with the goal of delaying progression and reducing morbidity and mortality (1).

There are currently no uniform definitions of cancer control and toxicity in the post-radiorecurrence setting, no randomized trials comparing any of local salvage therapies to ob-servation or ADT, and no analysis of the impact of pelvic lymph node dissection. At first glance, one might envisage a central role for the potentially less aggressive local approach offered by ablation strategies as opposed to salvage radical prostatectomy (SRP), mainly because if one has not opted for surgery as the first line treatment, the reasons against surgery will exponentially grow in the salvage setting.

However, in pooled head to head data, even based on the more permissive recurrence definitions of ablative therapies, ablative approaches do not necessarily supplant SRP in terms of oncological results and have also not always exhibited the expected dramatic decrease in morbidity when compared to SRP (2, 3).

As radiotherapy advances and improves over time, inadequate deliveries decrease and when necessary, SRP tends to be less challenging, as evidenced by improvement in surgical morbidity and oncologic outcomes in later series, especially in high volume centers where Clavien grade 3 or higher complications are uncommon and successfully treated in most cases (2). Also, small technical modifications such as anterior repositioning of the bladder neck outside the radiation field might lead to improved functional results.

A benefit of SRP is that it adds the clear goal of an undetectable PSA and the opportunity for pelvic lymph-node dissection (LND) to potentially treat loco regional micrometastasis and delay further progression, although cancer-specific survival data is still needed (4). Also, the particular pattern of tumor recurrence after RT in the periurethral zone (5) is often spared during certain ablative approaches such as HiFU and cryotherapy, excluding cancer foci in regions such as apex or periurethral tissue to minimize side effects.

Low-risk disease patients show higher chances of cancer control, but also carry a higher risk of overtreatment in the radiorecurrent setting. Identifying better markers of metastatic disease is critical to determining which patients are salvage treatment candidates and current rapidly-improving imaging tools impacting the accuracy to distinguish local from locoregional and distant recurrence play a central role.

Initial low-risk disease, long PSADT, low pre salvage PSA, and a lower Gleason score at the time of recurrence indicate a better likelihood of response to local treatment and both improved patient selection and exclusion of metastatic disease are fundamental, independent of therapeutic choice. While recently introduced whole-body MRI, PET with radiolabelled PSMA, bombesin or uPAR and PET/MRI might improve staging accuracy, no standard protocol has been shown to completely rule out the possibility of subclinical metastases (6).

To minimize overtreatment, patients should have: 1 - life expectancy long enough to benefit from intervention; 2 - a disease that is aggressive enough to warrant salvage therapy; 3 - but not so aggressive that there is a high chance that the patient has disseminated disease beyond the capacity of locoregional treatment for curative intent.

Pending high-quality randomized studies, the patient should be involved in an individualized decision-making process where all treatment options pros and cons should be discussed in a multi-disciplinary team and independent of salvage local treatment choice, patient should be highly motivated and aware of significant morbidity and risk of future multimodal treatment.
